# Translating Risk Ratios, Baseline Incidence, and Proportions Diseased to Correlations and Chi-Squared Statistics: Simulation Epidemiology

**DOI:** 10.7759/cureus.62769

**Published:** 2024-06-20

**Authors:** Yi-Sheng Chao, Chao-Jung Wu, Yi-Chun Lai, Hui-Ting Hsu, Yen-Po Cheng, Hsing-Chien Wu, Shih-Yu Huang, Wei-Chih Chen

**Affiliations:** 1 Epidemiology and Public Health, Independent Researcher, Montreal, CAN; 2 Computer Sciences, Université du Québec à Montréal, Montreal, CAN; 3 Chest Medicine, National Yang Ming Chiao Tung University Hospital, Yilan, TWN; 4 Pathology, Changhua Christian Hospital, Changhua, TWN; 5 Neurological Surgery, Changhua Christian Hospital, Changhua, TWN; 6 Internal Medicine, National Taiwan University Hospital, Taipei, TWN; 7 Anesthesiology, Taipei Medical University Shuang-Ho Hospital, New Taipei, TWN; 8 Chest Medicine, Taipei Veterans General Hospital, Taipei, TWN

**Keywords:** incidence, chi-squared statistics, correlation, risk ratio, measures of association

## Abstract

Background

In a population, when a disease is causing a symptom, the overall symptom incidence can be determined by proportions diseased, baseline symptom incidence, and risk ratios of developing the symptom due to the disease. There are various measures of association, including risk ratios. How risk ratios are linked to other measures of association, such as correlation coefficients and chi-squared statistics, has not been explicitly discussed. This study aims to demonstrate their connection via equations and simulations, assuming one disease causes symptoms.

Methods

The equations for correlation coefficients and chi-square statistics were rewritten using epidemiological measures: proportions diseased, baseline symptom incidence, and risk ratios. Simulations were conducted to test the accuracy of the equations. The baseline symptom incidence and the proportions diseased were assumed to be 0.05, 0.1, 0.2, 0.4, or 0.8. The risk ratios were assumed to be 0.5, 1, 2, 5, 10, and 25. Another disease that correlates with this disease was created (correlation = 0, 0.3, or 0.7). For each combination of symptom incidence, proportions diseased, risk ratios, and between-disease correlations, 10,000 subjects were simulated. The correlation coefficients and chi-squared statistics were approximated with epidemiologic measures and their interaction terms. R-squared was used to assess the importance of the epidemiologic measures.

Results

In the simulations, the overall symptom incidence, correlation coefficients, and chi-squared statistics between the disease and symptoms could be fully explained by the epidemiologic measures in the equations (R-squared = 1). When approximating correlation coefficients and chi-squared statistics with individual measures or their interaction terms, the importance of these measures depended on whether the at-risk incidence reached 1 or not. The numbers in the four cells in the contingency table predicted correlation coefficients, or chi-squared statistics, with different R-squared.

Conclusion

To our knowledge, this is the first study to translate the three epidemiologic measures (risk ratios, baseline symptom incidence, and proportions diseased) into correlation coefficients and chi-squared statistics. However, chi-squared statistics also depend on sample sizes. This study also provides a platform for developing teaching cases for students to investigate the causal relationship between diseases and symptoms or exposure and outcomes.

## Introduction

Various measures of association have been used to quantify the relationships between risk factors and outcomes [1,2]. For example, risk ratios are relative measures of risk factors that should be interpreted with the baseline incidence among those not at risk [3]. Risk ratios have been widely used and regarded as a standard measure to report in cohort studies or clinical trials, denoted by *rr* [4,5]. Risk ratios are calculated by dividing the cumulative incidence of developing symptoms among those at risk or diseased by the baseline cumulative incidence, denoted by *ir*, among those not at risk [4,5]. In a population whose symptoms can occur randomly and the risk of developing symptoms is proportional to the disease status, the overall incidence can be derived if the proportions of being at risk, denoted by *pr*, are known: [*pr×ir×rr+(1-pr)×ir*]. In a simplistic model where symptoms develop randomly and become more common among those who are diseased, these epidemiologic measures can determine the overall incidence of the symptoms. These measures can be effectively used to describe how diseases are related to symptoms. However, how these epidemiologic measures are linked to other measures of association is not clear.

Other measures of association, including correlation coefficients and chi-square statistics, are often used to describe the strengths of associations in related settings [3]. Pearson correlation coefficients quantify the relationships between two variables, including binomial variables [3]. The range of correlation coefficients is between -1, which is completely opposite, and 1, which is exactly the same [3]. Chi-square tests examine the association between two categorical variables, depending on the sample size [3]. Mathematically, these measures can be linked with the above-mentioned epidemiological measures. The overall incidence of the symptoms, [*pr×ir×rr+(1-pr)×ir*], should be proportional to the proportions diseased, *pr*, and the risk ratio of symptom occurrence, *rr*.

For chi-square statistics used to assess the disease prevalence and the occurrence of its symptoms in a 2-by-2 contingency table, the increases in the risk ratios and baseline symptom cumulative incidence can lead to uneven distributions of sample sizes in the cells and various chi-squared statistics [6].

In biological research, there are often animal or cell models built in laboratories for researchers to demonstrate biological pathways between exposure and outcome [7,8]. The discussion on “ideal” or “laboratory” scenarios is increasing for epidemiologists. Though it is unclear how different epidemiologic and statistical measures can be connected in an ideal scenario, we have experience using simulation epidemiology to demonstrate the biases embedded in the diagnostic criteria for mental illnesses and the upper limits of risk ratios [2,9]. Other researchers used simulation for epidemiological education, research, and prediction [1,10,11]. We think it is important to build teaching examples that link various measures and can be used as a reference for researchers to assess whether the analysis of real-world data matches simulated results. In this study, we aim to use simulations to determine the relationships between various measures of association, including risk ratios, correlations, and chi-squared statistics.

## Materials and methods

**Figure 1:**
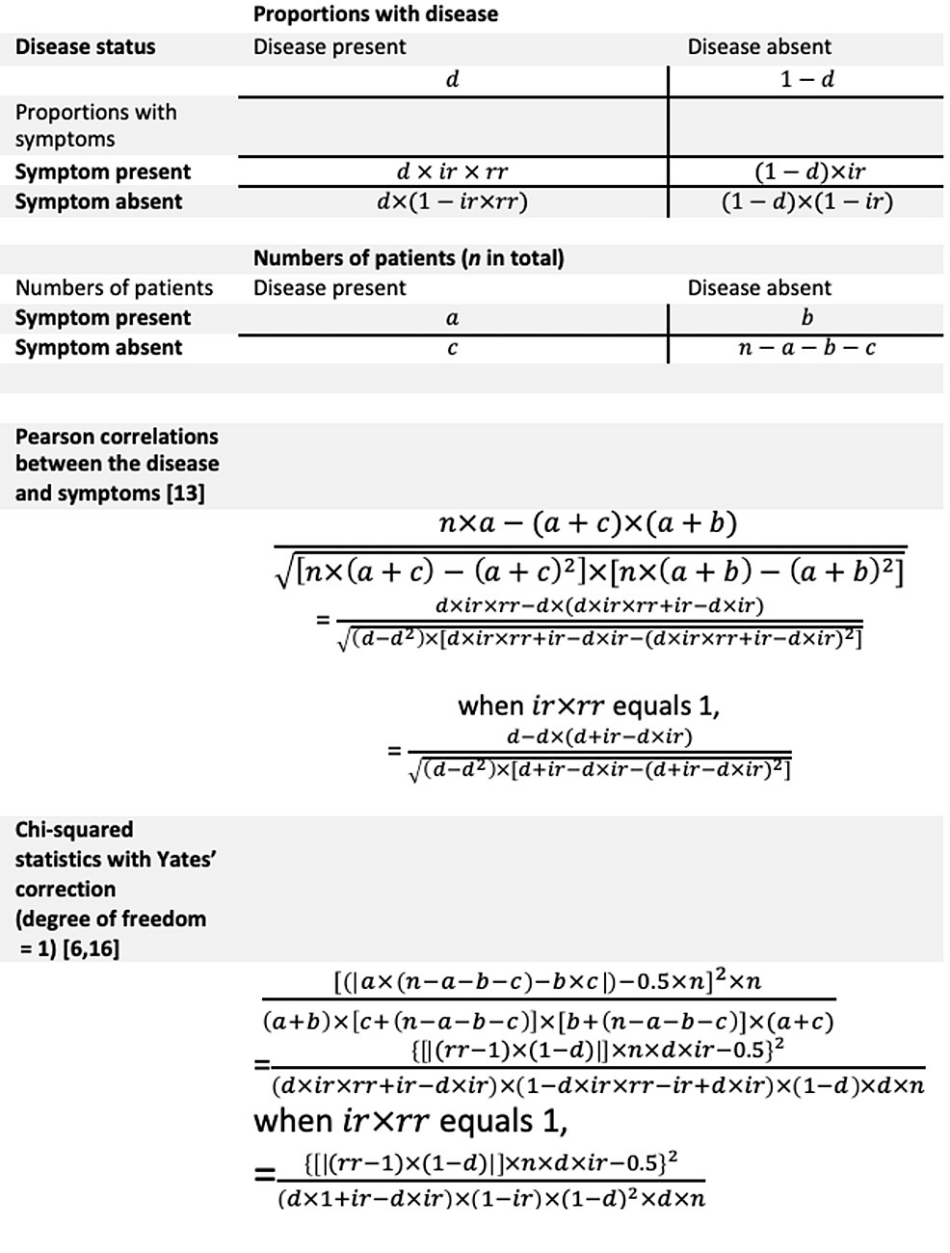
Translating proportions diseased, baseline incidence, and risk ratios to correlation coefficients and chi-squared statistics *a* = the number of subjects with the disease and symptom; *b* = the number of subjects with the symptom only; *c* = the number of subjects with the disease only; *d* = the proportion in the population with the disease; *n* = the number of all subjects; *ir* = baseline incidence rates among those without the disease; *rr* = risk ratios of developing the symptom due to the disease

The study was conducted by Yi-Sheng Chao in an independent research institute in Montreal, Canada. In Figure 1, the correlations between the disease and its symptoms are derived based on the baseline cumulative incidence of the symptoms, proportions diseased, and risk ratios. In Figure 1, chi-squared statistics are sensitive to overall sample sizes and the changes in the proportions diseased, risk ratios, and baseline incidence. However, how various measures of association are related has not been explicitly explored to our knowledge.

**Figure 2:**
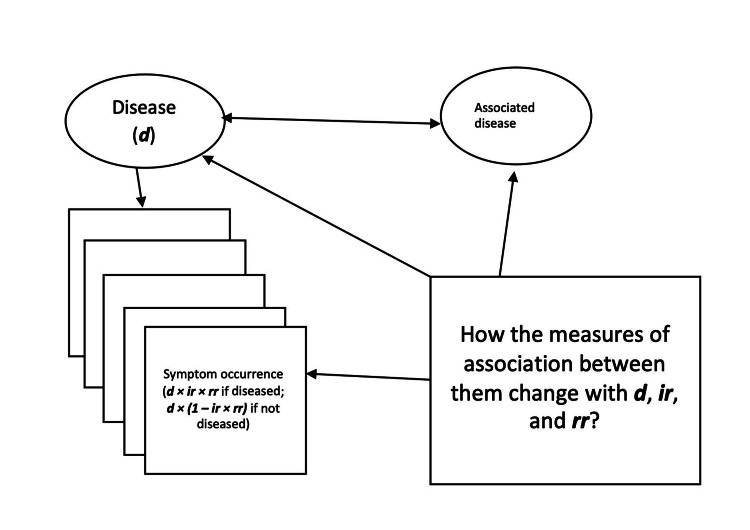
The elements of the simulations *d*: proportions diseased in a population; *ir*: baseline incidence rates among those not diseased; *rr*: risk ratios among those diseased. The measures of association studied include correlations and chi-squared statistics.

The assumptions and the epidemiologic measures used for simulations are listed in Table 1. In detail, there were only two diseases assumed in Figure 2. One directly influenced the incidence of the symptoms. The other disease was associated with the symptom-causing disease only. A symptom developed based on the assumed baseline cumulative incidence and risk ratios. For both diseases and symptoms, a value of 1 represented the presence of diseases or symptoms, and a value of 0 represented their absence. The products of the incidence rates and risk ratios could not exceed 1, a maximum of 100% incidence rate among those with or without the disease [12]. The Pearson correlation coefficients between the disease and the other associated disease were 0, 0.3, or 0.7. The prevalence rates of the disease or proportions diseased (*d*) were assumed to be 0.05, 0.1, 0.2, 0.4, or 0.8. The baseline cumulative incidence (*ir*) of developing symptoms for those not affected by the disease was 0, 0.1, 0.2, 0.4, and 0.8. The risk ratios (*rr*) of developing symptoms if diseased were 0.5 (less likely to develop symptoms), 1.0 (equally likely to develop symptoms), 2.0, 5.0, 10.0, or 25.0 (more likely to develop symptoms). There were 10,000 individuals simulated for each combination of the above-mentioned epidemiologic measures. For each combination of the epidemiologic measures, there were 10 simulations to obtain statistics.

**Table 1:** The assumptions and the assessments of the simulated symptoms

	Assumptions	
1	Two diseases of interest: one disease directly causing the symptoms and the other associated with the disease only (unrelated to the symptoms)	
2	Accurate disease statuses and symptoms reported accurately by patients	
3	The products of incidence rates and risk ratios less than or equal to 1	
4	Symptoms occur randomly based on the incidence	
	Parameters of symptom occurrence simulations	
1	Population sizes (n)	10,000
2	Correlation between diseases	0, 0.3, or 0.7
3	Prevalence rates of the diseases or proportions diseased (d)	0.05, 0.1, 0.2, 0.4, or 0.8
4	Cumulative incidence rates of symptoms (ir)	0.05, 0.1, 0.2, 0.4, or 0.8
5	Risk ratios of developing symptoms if diseased (rr)	0.5, 1, 2, 5, 10, or 25
6	Number of simulations for each combination of Parameter 2 to 5	10
	Statistics for assessment	
	Correlations between the symptoms and the disease	
	Chi-square statistics between the symptoms and the disease	

Correlation coefficients

Pearson's correlation coefficients are a measure of linear association [13-15]. The equations to derive correlation coefficients based on baseline incidence, risk ratios, and proportions diseased are listed in Figure 1. The statistical significance of the correlation coefficients was also obtained [13]. The baseline incidence, risk ratios, and proportions diseased were used as independent variables in linear regression models to understand their role in correlation coefficients and statistical significance (p values). In addition, there were other interaction terms selected based on the equations in Figure 1 to approximate measures of associations.

Chi-square statistics

Chi-squared statistics were calculated based on the expected and observed numbers in a contingency table that demonstrates the distributions of a disease and a symptom [6,16]. Therefore, chi-squared statistics depended on the sample sizes [6]. The equations for derived chi-squared statistics based on baseline cumulative incidence, risk ratios, and proportions diseased are listed in Figure 1.

R-squared that showed the proportions of dependent variable variances explained by independent variables were extracted when approximating correlation coefficients or chi-squared statistics [17]. P values that were two-tailed and less than 0.05 were considered statistically significant. Multicollinearity was evaluated with variance inflation factors [18]. A variance inflation factor larger than 10 was considered problematic [18]. All statistical analyses were conducted under the R environment (v3.5.1, Vienna, Austria) [19] and RStudio (v1.1.463, RStudio, Inc., Boston, MA) [20]. R codes are available in the supplemental materials.

## Results

Derived and assumed values

The relationship between assumed and derived baseline cumulative incidence and risk ratios of the symptoms is presented in Appendix 1. In brief, the derived incidence among those not at risk was similar to the assumed values, suggesting successful simulations as shown in Appendix 2. However, there were upper limits to the risk ratios (the multiplicative inverse of the baseline cumulative incidence among those not at risk) [9]. In the simulations in which those at risk all presented symptoms with an assumed 0.05, 0.1, 0.2, 0.4, or 0.8 baseline incidence among those not diseased, the upper limits of the risk ratios are around 20, 10, 5, 2.5, or 1.25 [9]. When the derived risk ratios did not exceed these upper limits, the derived risk ratios were similar to the assumed values. The implementation of the simulations was acceptable.

Symptom incidence

The overall incidence was related to the assumed values of baseline incidence, proportions at risk, and risk ratios in Figure 1. The derived overall incidence could be fully predicted by baseline incidence and two interaction terms: the interaction between derived risk ratios, observed baseline incidence, and proportions at risk, and the other interaction term between observed incidence and proportions at risk (R-squared = 1).

Correlation between disease and symptom

**Figure 3:**
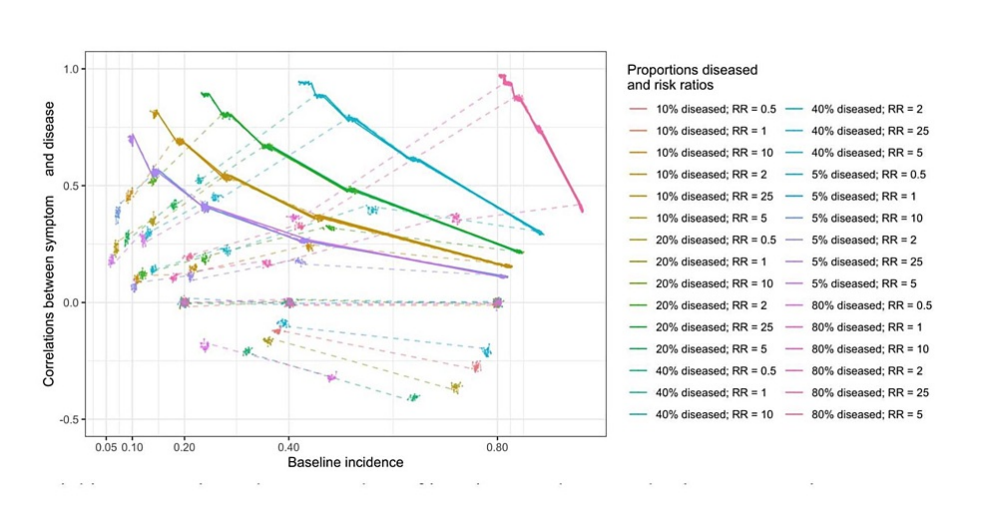
The relationship between correlation coefficients and epidemiologic measures: proportions diseased, risk ratios, and baseline incidence Solid lines: at-risk incidence, product of baseline incidence and risk ratios, reaching 1; dashed lines: at-risk incidence less than 1

The correlation coefficients were fully predicted by all the terms in the equations in Figure 1 (R-squared = 1, regression coefficients not shown). When approximating the correlation coefficients with selected epidemiologic measures, baseline symptom incidence, risk ratios, and proportions diseased were significantly associated with the correlation coefficients in Table 2. Overall, the variances of correlation coefficients could be explained by the three epidemiologic measures, R-squared, being 0.53 and 0.94 when the at-risk incidence (product of baseline incidence and risk ratios) was less than 1 and reaching 1, respectively. In Figure 3, the relationship between correlation coefficients and epidemiologic measures is illustrated. When the at-risk incidence was less than 1, proportions diseased and risk ratios were proportional to the correlation coefficients. Symptom incidence was not proportional to the correlation coefficients. When the at-risk incidence reached 1, the effect sizes of these three measures changed, but the directions remained the same.

**Table 2:** Approximating correlation coefficients with epidemiologic measures and their interaction terms Assumptions listed in Table 1

	At-risk incidence less than 1						At-risk incidence reaching 1					
	Coefficients	(95% CIs)	P	Coefficients	(95% CIs)	P	Coefficients	(95% CIs)	P	Coefficients	(95% CIs)	P
Intercept	0.06	(0.06 to 0.06)	0	-0.01	(-0.01 to -0.01)	0	0.6	(0.59 to 0.60)	0	10583.46	(7263.99 to 13902.94)	0
Correlation between diseases	0	(0.00 to 0.00)	0.45	0	(0.00 to 0.00)	0.55	0	(0.00 to 0.00)	0.04	0	(0.00 to 0.00)	0.02
Proportions diseased	0.18	(0.17 to 0.18)	0	0.03	(0.03 to 0.03)	0	0.47	(0.47 to 0.47)	0	0.78	(0.78 to 0.79)	0
Risk ratios	0.05	(0.05 to 0.05)	0	0	(0.00 to 0.00)	0	0.01	(0.01 to 0.01)	0	0.02	(0.02 to 0.02)	0
Baseline symptom incidence	-0.11	(-0.11 to -0.10)	0	-0.82	(-0.82 to -0.81)	0	-0.64	(-0.65 to -0.64)	0	-0.5	(-0.51 to -0.50)	0
Interaction terms												
Baseline symptom incidence X Risk ratios				0.78	(0.78 to 0.78)	0				-10582.97	(-13902.44 to -7263.49)	0
Proportions diseased X Risk ratios				0.02	(0.02 to 0.02)	0				-0.03	(-0.03 to -0.02)	0
Proportions diseased X Baseline symptom incidence				0.22	(0.21 to 0.22)	0				-0.44	(-0.45 to -0.44)	0
R-squared	0.53			0.93			0.94			0.95		
Degrees of freedom	314734			314734			117264			117264		

When selected interaction terms were added, the variances of correlation coefficients could be explained by epidemiologic measures, R-squared, increasing to 0.93 and 0.95 when at-risk incidence was less than 1 and reaching 1, respectively, in Table 2. However, the direction of proportions diseased changed and became disproportional to correlation coefficients when the at-risk incidence was less than 1. When at-risk incidence reached 1 and the interaction terms were added, the regression coefficient of the intercept became the largest (10,583.46, p<0.05), and the interaction between baseline symptom incidence and risk ratios also had a large regression coefficient in the opposite direction (-10,582.97, p<0.05).

Chi-squared statistics between the disease and the symptom

**Figure 4:**
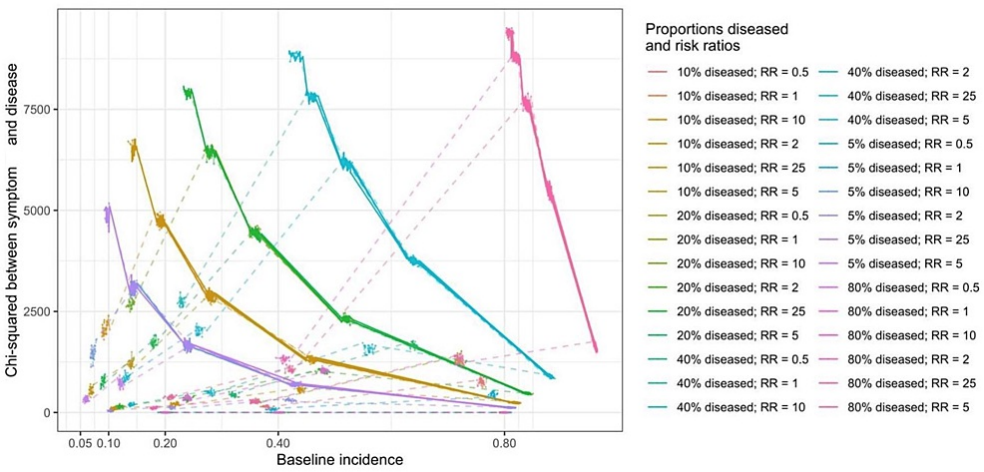
The relationship between chi-square statistics and epidemiologic measures: proportions diseased, risk ratios, and baseline incidence Solid lines: at-risk incidence, product of baseline incidence and risk ratios, reaching 1; dashed lines: at-risk incidence less than 1

In Figure 1, chi-squared statistics depended on sample sizes. In the simulations with 10,000 individuals, the chi-squared statistics to assess the associations between the disease and symptoms were fully predicted by all the terms in the equations in Figure 1 (R-squared = 1, regression coefficients not shown). When approximating the chi-squared statistics with selected epidemiologic measures, proportions diseased, risk ratios, and baseline symptom incidence significantly predicted the chi-squared statistics in Table 3. The proportions of the variances of chi-squared statistics explained by the epidemiologic measures, R-squared, were 0.56 and 0.91 when at-risk incidence was less than 1 and reaching 1, respectively. In Figure 4, the relationship between chi-squared statistics and epidemiologic measures is shown. When the at-risk incidence was less than 1, proportions diseased, risk ratios, and baseline symptom incidence were proportional to chi-squared statistics. When the at-risk incidence reached 1, proportions diseased and risk ratios were proportional to chi-squared statistics, but baseline symptom incidence was not.

**Table 3:** Approximating chi-squared statistics with epidemiologic measures and their interaction terms Assumptions listed in Table 1

	At-risk incidence less than 1						At-risk incidence reaching 1					
	Coefficients	(95% CIs)	P	Coefficients	(95% CIs)	P	Coefficients	(95% CIs)	P	Coefficients	(95% CIs)	P
Intercept	-872.42	(-888.33 to -856.52)	0	-884.13	(-901.06 to -867.20)	0	3168.53	(3149.59 to 3187.46)	0	137354153.6	(100547517.50 to 174160789.75)	0
Correlation between diseases	0.39	(-17.85 to 18.63)	0.97	-4.74	(-19.12 to 9.63)	0.52	-9.37	(-26.60 to 7.87)	0.29	-10.54	(-23.00 to 1.91)	0.1
Proportions diseased	2042.2	(2023.08 to 2061.31)	0	395.6	(356.77 to 434.44)	0	5189.76	(5171.66 to 5207.86)	0	10924.13	(10878.92 to 10969.35)	0
Risk ratios	425.79	(424.21 to 427.37)	0	142.87	(140.79 to 144.95)	0	162.52	(161.13 to 163.92)	0	250.72	(249.18 to 252.25)	0
Baseline symptom incidence	938.88	(915.56 to 962.21)	0	-2403.73	(-2434.64 to -2372.82)	0	-5469.32	(-5494.86 to -5443.78)	0	-2160.16	(-2188.92 to -2131.40)	0
Interaction terms												
Baseline symptom incidence X Risk ratios				3481.27	(3464.92 to 3497.63)	0				-137352761.5	(-174159395.81 to -100546127.19)	0
Proportions diseased X Risk ratios				301.64	(297.10 to 306.18)	0				-280.79	(-284.46 to -277.12)	0
Proportions diseased X Baseline symptom incidence				1550.56	(1483.27 to 1617.85)	0				-10584.43	(-10651.89 to -10516.97)	0
R-squared	0.56			0.73			0.91			0.95		
Degrees of freedom	314734			314734			117264			117264		

When the selected interaction terms were added for the prediction of chi-squared statistics, the R-squared increased to 0.73 and 0.95 when at-risk incidence was less than 1 and reaching 1, respectively, in Table 3. The regression coefficients of baseline symptom incidence became negative (-2,403.73), and those of proportions diseased and risk ratios remained in the same directions (395.60 and 142.87, respectively) when the at-risk incidence was less than 1 and the interaction terms were added. When interaction terms were added and the at-risk incidence reached 1, the regression coefficients remained in the same direction. However, the regression coefficients of the intercept increased to a large number (137,354,153.63, p<0.05), and the interaction term between baseline symptom incidence and risk ratios had a negative regression coefficient of similar size (-137,352,761.50, p<0.05).

Correlation and chi-squared statistics 

**Figure 5:**
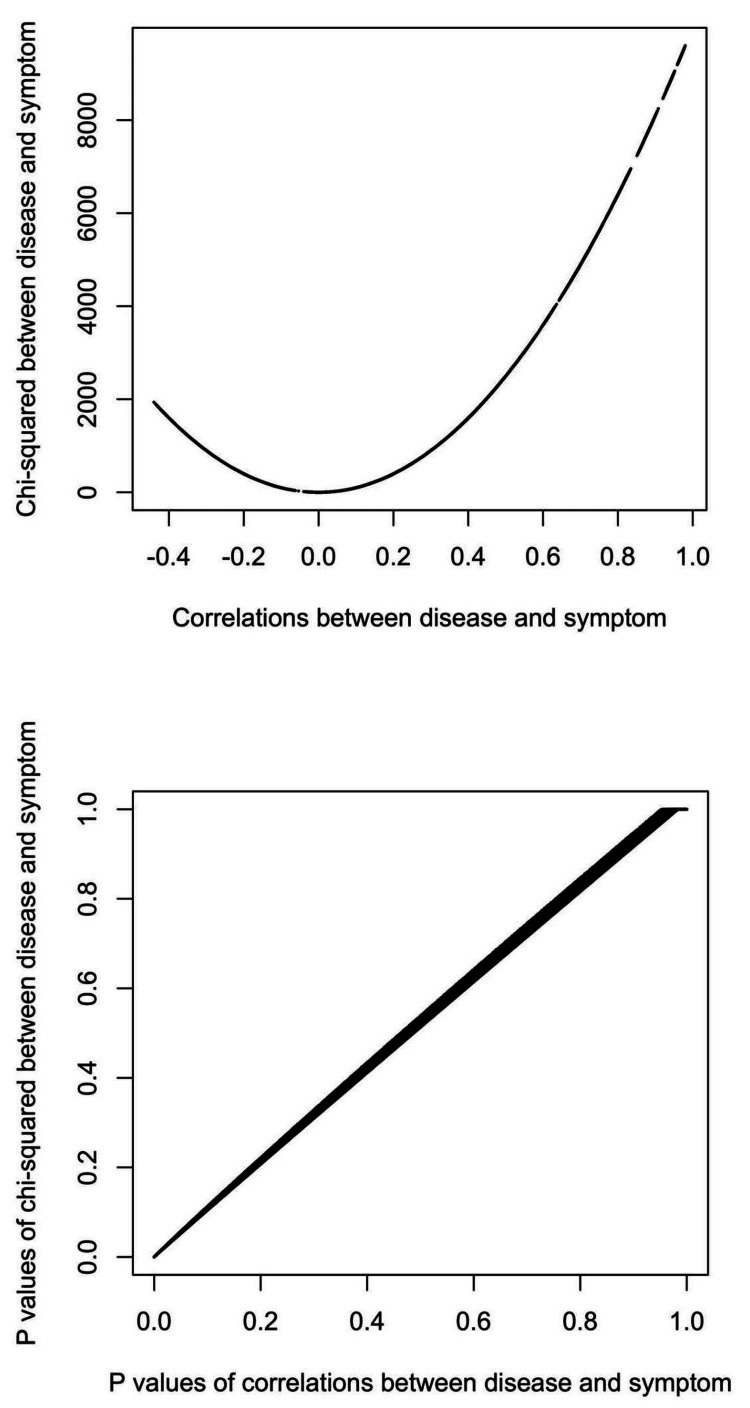
The relationship between correlation coefficients and chi-squared statistics

The chi-squared statistics were proportional to the absolute values of the correlation coefficients in Figure 5. The p values of chi-squared statistics were linearly proportional to those of correlation coefficients. Among 432,000 simulations, the p values of both chi-squared statistics and correlation coefficients were significant and insignificant in 380,398 and 51,396 (88.1% and 11.9%, respectively) simulations. The p values of correlation coefficients were significant in 206 (0.05%) simulations that had insignificant chi-squared p values.

R-squared for the prediction of correlation coefficients and chi-squared statistics

**Figure 6:**
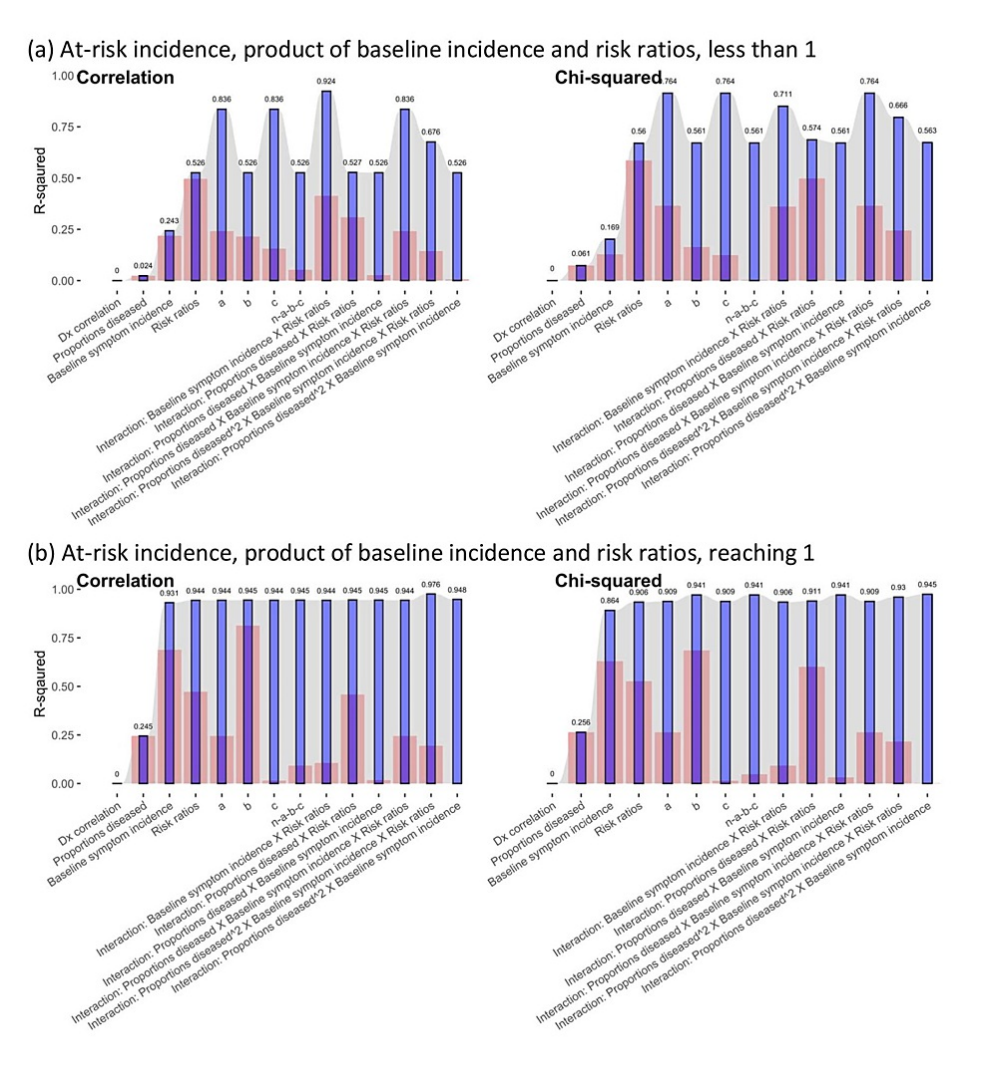
R-squared of epidemiologic measures for the prediction of the correlation coefficients and chi-squared statistics between a disease and its symptoms *a, b, c*, and *n-a-b-c* = numbers of individuals in Figure 1. Dx correlation: correlations between diseases Red bars: R-squared of single variables for the prediction of the correlation coefficients or chi-squared statistics between a disease and its symptoms; Blue bars: R-squared of the variable and those listed to its left for the prediction of the correlation coefficients or chi-squared statistics between a disease and its symptoms. The epidemiologic measures, correlations between diseases, proportions diseased, baseline symptom incidence, and risk ratios were sequentially added to regression models for assessing the increase in R-squared regarding correlation coefficients or chi-squared statistics. The other variables were separately and individually added to regression models with these four measures to identify the increase in R-squared.

In addition to epidemiologic measures, the numbers of individuals in the four cells in the contingency tables, denoted by a, b, c, and n-a-b-c in Figure 1, also mattered. When assessed using R-squared, the importance of epidemiologic measures and the numbers of individuals in the four cells were compared based on how well they predicted correlation coefficients and chi-squared statistics in Figure 6, individually or in combination with epidemiologic measures.

Individually, the numbers of individuals in the four cells, *a, b, c,* and *n-a-b-c*, had different predictive power for these two measures of association. Their predictive power also depended on whether the at-risk incidence, a product of the baseline symptom incidence and risk ratios, reached 1. The numbers of individuals with symptoms, whether diseased (*d*) or not (*1-d*), individually predicted the correlation coefficients and chi-squared statistics better than the other two (red bars in Figure 6). The correlations between diseases did not significantly explain the correlation coefficients or chi-squared statistics between the disease and its symptoms (regression coefficients in Table 2 and Table 3; R-squared in Figure 6).

Among the interaction terms, when the at-risk incidence was less than 1, the interaction between baseline symptom incidence and risk ratios, the interaction between proportions diseased and risk ratios, and the interaction between proportions diseased, baseline symptom incidence, and risk ratios could reach more than 0.25 R-squared for explaining correlation coefficients or chi-squared statistics. When the at-risk incidence reached 1, the interaction between proportions diseased and risk ratios individually explained correlation coefficients, or chi-squared statistics, the best in terms of R-squared.

Collectively, the epidemiologic measures, correlations between diseases, proportions diseased, baseline symptom incidence, and risk ratios were sequentially added to regression models for assessing the increase in R-squared regarding correlation coefficients or chi-squared statistics in Figure 6. The addition of these measures led to increases in the R-squared for explaining correlation coefficients, or chi-squared statistics (blue bars in Figure 6). The other variables were separately and individually added to regression models with these four measures to identify the increase in R-squared.

When the at-risk incidence was less than 1, the epidemiologic measures together could lead to 0.526 and 0.560 R-squared for correlation coefficients and chi-squared statistics, respectively. However, adding several variables or interaction terms did not lead to a major increase in R-squared, such as *b, n-a-b-c,* the interaction between proportions diseased and risk ratios, the interaction between propositions diseased and baseline symptom incidence, and interaction between square proportion diseased and baseline symptom incidence. Adding the interaction between baseline symptom incidence and risk ratios could lead to the largest increase in R-squared for correlation coefficients. Adding the interaction between proportion diseased, baseline symptom incidence, and risk ratios could lead to the largest increases in R-squared for chi-squared statistics.

When the at-risk incidence reached 1, the epidemiologic measures together could lead to 0.944 and 0.906 R-squared for correlation coefficients and chi-squared statistics, respectively. Adding other variables could increase the R-squared but to a lesser extent than when the at-risk incidence was less than 1.

## Discussion

It is easy to understand that different measures of association are related. However, no matter how large the sample size is in a clinical study, researchers can only observe a risk ratio or a correlation coefficient based on a disease and a symptom [9]. It is rare to observe multiple risk ratios or correlation coefficients based on a disease and a symptom in a homogenous and well-defined population. It is difficult, and there are few opportunities to translate a large number of epidemiological measures into measures of association with comparable populations in the real world. Simulation epidemiology helps researchers obtain large numbers of epidemiological measures and illustrate the relationships between different measures of association [15,21].

Risk ratios of developing symptoms among those diseased, a measure of association, can be translated to correlation coefficients and chi-squared statistics with proportions diseased, baseline symptom incidence (for both), and sample sizes (for chi-squared statistics only). In the simulations, epidemiologic measures can be converted to 2-by-2 tables and subsequently correlation coefficients and chi-squared statistics according to the equations in Figure 1. In textbooks, measures of association might not be taught together, and the link between them is not explicitly demonstrated [22,23]. This is the first study to link the three measures of association to our knowledge.

Overall, there is an upper limit to the at-risk incidence, a product of baseline incidence and risk ratios: 1 [9]. The role of the epidemiologic measures (proportions diseased, baseline symptom incidence, and risk ratios) on the measures of association depended on whether the at-risk incidence reached 1. Moreover, the importance of various epidemiologic measures has been assessed and compared with each other for their contribution to the statistical measures of association. There are several interaction terms identified from the equations and assessed for their role in correlation coefficients and chi-squared statistics.

When the at-risk incidence does not reach 1, risk ratios predict correlation coefficients and chi-squared statistics the best. When the at-risk incidence reaches 1, the baseline incidence predicts them the best. Proportions diseased predict them the least, with at-risk incidence reaching 1 or not. We did not find relevant studies that show similar findings.

Key interaction terms

There are several key interaction terms identified in the equations and examined for their role in predicting measures of association. With at-risk incidence reaching 1 or not, the interaction between baseline incidence and risk ratios predicted correlation coefficients better, and the interaction between proportions diseased and risk ratios predicted chi-squared statistics better. However, the magnitude of their impact is modified by whether at-risk incidence reaches 1. We did not find major studies on these interaction terms and will continue studying their role in other measures of association.

Number of individuals in the contingency table

Unexpectedly, the numbers of individuals in the contingency tables do not have the same importance for correlation coefficients or chi-squared statistics. Traditionally, the numbers of individuals in the four cells in the contingency tables are not treated differently [22]. Based on how they are derived from epidemiologic measures, they are, in fact, complicated interaction terms in Figure 1. Our findings show that these four numbers may explain the associations between diseases and symptoms better than the three epidemiologic measures and simpler interaction terms listed in Figure 6, especially when at-risk incidence reaches 1. To our knowledge, this is the first study to show the difference in their contribution to measures of association.

Association between diseases

The association between diseases does not influence the association between the disease and its symptoms. The correlations between diseases do not significantly explain the correlation coefficients and chi-squared statistics between the disease and its symptoms. The association between diseases has not confounded the relationship between the disease and its symptoms, and the simulations did not introduce this related disease as a confounder that should be directly associated with both the disease and its symptoms [22]. The related disease does not significantly influence the correlation coefficients or chi-squared statistics between the disease and its symptoms in Figure 6. In addition, adding a related disease that did not cause undesirable confounding helps us to confirm the usefulness of the simulations and that the causal relationship between the disease and symptoms was not influenced.

Future research directions

There are other measures of association to be linked. For example, there are statistical measures of association, such as phi and contingency coefficients [24,25]. There are epidemiologic measures of association, such as odds ratios and incidence rate ratios [22,26]. There are other variable types for assessment, such as interval and ordinal variables [27,28]. We think there are a lot of topics for researchers. Based on the simulations in this study, we attempted to build a teaching example for students to illustrate causal relationships and explore other issues for research. Furthermore, by searching for real-world cases that violate the predictions from laboratory models, we may have better clues about the causal relationships between risks and outcomes or diseases and their symptoms.

Limitations

This study links epidemiologic measures and measures of association that are familiar to researchers. There are limitations to this study. The assumptions of random assignment of symptoms based on incidence may not be realistic in a 10,000-person population. Other factors that may influence the relationship between statistical and epidemiologic measures of association may not be captured in the equations, for example, heterogeneity in the population characteristics. In a population that has a wide age range, symptoms may concentrate among the elderly or children in the real world. It takes caution to generalize the relationship between different measures of association. However, we think this is also a research opportunity that we are exploring.

## Conclusions

We think this is the first study to translate three epidemiologic measures: risk ratios, baseline symptom incidence, and proportions diseased, into other measures of association, Pearson’s correlation coefficients, and chi-squared statistics. Risk ratios, along with other measures, can be translated to other measures of association, particularly correlation coefficients and chi-squared statistics, in a model where one disease directly causes symptoms. Mathematically, correlation coefficients do not depend on sample sizes, but chi-squared statistics vary partly based on sample sizes. Moreover, there are upper limits to risk ratios, the multiplicative inverse of the baseline incidence. When translating risk ratios to correlation coefficients and chi-squared statistics, it is important to consider whether the at-risk incidence, product of risk ratios, and baseline symptom incidence may reach 1. Using this one-disease-one-symptom model to simulate populations, the absolute values of correlation coefficients are proportional to chi-squared statistics. There are slightly more significant correlation coefficients than significant chi-squared statistics. When approximating correlation coefficients and chi-squared statistics using the three epidemiologic measures, the R-squared is less than 0.6, with an at-risk incidence less than 1. When interaction between epidemiologic measures is added, R-squared does not necessarily increase. The numbers of individuals in the contingency tables explain correlation coefficients and chi-squared statistics differently. The numbers of individuals with symptoms, diseased or not, explained correlation coefficients, or chi-squared statistics, better than the numbers without symptoms. We think the connection between epidemiologic measures and measures of association is well demonstrated with equations and simulation results. This study provides a platform for developing teaching cases for students to investigate the causal relationship between diseases and symptoms or exposure and diseases.

## Figures and Tables

**Figure 7:**
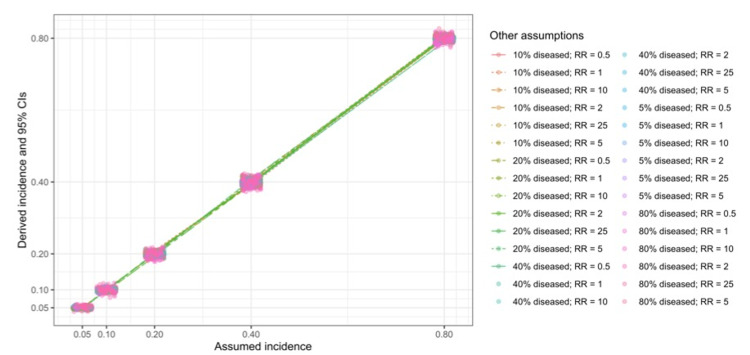
Assumed and derived incidence of the symptoms

**Figure 8:**
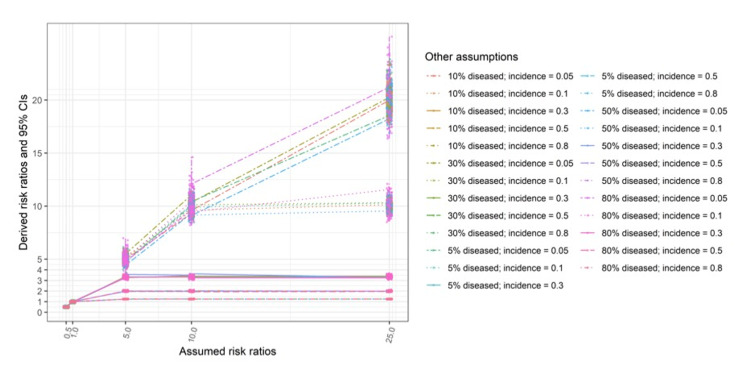
Assumed and derived risk ratios

## Appendices

Appendix 1

R codes are available upon request to the authors of this article.

Appendix 2
